# Perceptions of COVID-19 among frontline health workers in Burkina Faso

**DOI:** 10.3389/fsoc.2022.963817

**Published:** 2023-01-11

**Authors:** Fadima Yaya Bocoum, Kadidiatou Kadio, Télesphore Some, Adidjata Ouedraogo, Maxime Drabo, Seni Kouanda

**Affiliations:** ^1^Département Biomédical et Santé Publique, Institut de Recherche en Sciences de la Santé, Ouagadougou, Burkina Faso; ^2^African Population Health Research Center, WARO, Dakar, Senegal; ^3^Fellow Pilote African Postdoctrorat Academy - PAPA, Goethe University Frankfurt, Frankfurt, Germany; ^4^Institut Africain de Santé Publique (IASP), Ouagadougou, Burkina Faso

**Keywords:** perceptions, COVID-19, frontline, health personnel, Burkina Faso

## Abstract

**Introduction:**

In Burkina Faso, the first cases of COVID-19 were reported in March 2020. Health personnel are on the front line of COVID-19 control, and it is important to understand their perceptions and knowledge of the disease. The objective was to determine the knowledge and perceptions of healthcare personnel of COVID-19 in the city of Ouagadougou.

**Method:**

The study was conducted in five plots in the city of Ouagadougou. A total of 20 health workers from public and private health centers in the city of Ouagadougou were selected during May 2020. In-depth individual interviews were conducted, and a thematic analysis was performed using NVIVO.

**Results and discussion:**

The routes of transmission identified were promiscuity, respiratory tract, and physical contact. Various symptoms have been noted, such as fever, cough, and runny nose. However, they recognize that these symptoms are not sufficient to make a diagnosis of COVID-19. Similarly, the treatments mentioned are modern medicine and phytomedicine.

**Conclusion:**

The research has generated information on healthcare workers' knowledge and perceptions of COVID-19. Therefore, they are useful for decision-making regarding protective measures for health workers in the management of COVID-19.

## Introduction

Between January and April 2020, the COVID-19 epidemic developed into a global pandemic from Wuhan, China, to reach the largest number of countries on all five continents (WHO, 2020). As of October 15, 2020, there were a cumulative 38,686,505 confirmed cases with 1,094,381 deaths worldwide (Dong et al., 2020).

Healthcare workers are on the front lines of the fight against COVID-19 in all countries (OMS, 2020). This means that healthcare workers, especially those who are in contact with or provide care to patients with COVID-19, are at greater risk of infection with SARS-CoV-2 than the general population (Word Health Organization, 2020). Knowledge can influence healthcare professionals through adoption of the correct attitudes and practices (Arrais et al., 2022).

Various studies have been conducted in different countries (Burkina Faso, Senegal, Niger) on knowledge, perceptions, and beliefs about COVID-19. Most of these studies were related to the general population or certain categories, such as the young and the elderly (Mahmood et al., 2020; Malo et al., 2020; Attema et al., 2021; Mathonnat et al., 2021; Hensel et al., 2022).

But there are studies conducted in sub-Saharan African countries using quantitative approaches that have assessed the level of knowledge, attitudes, and practices of health care workers regarding COVID-19 (Abu et al., 2021; Assefa et al., 2021; Ekpenyong et al., 2021; Feldman et al., 2021; Abduljaleel et al., 2022; Arrais et al., 2022; Benjamin et al., 2022; Olateju et al., 2022). Arrais et al. (2022) showed that the main concerns of physicians were training opportunities and limited access to personal protective equipment.

The WHO in its guidelines advocates for ensuring that sufficient staffing is maintained and that health personnel are properly trained in infection control measures for infection control programs to prove effective in preventing nosocomial infections, including during outbreaks (OMS, 2017). This is especially important because a study conducted in Burkina Faso found that government and health personnel were the main sources of information and the ones that community members trusted most to learn about COVID-19 (Ground Truth Solutions, 2020). In addition, knowledge and perception of a disease affected the management of the disease. Therefore, it is important to understand the perceptions of frontline health personnel about COVID-19 to improve the management of the disease and indirectly the population perceptions. Lack of knowledge could delay recognition and handling of potential COVID-19 patients (Bhagavathula et al., 2020).

The objective of this study was to explore the perceptions and knowledge of frontline health workers about COVID-19 in Burkina Faso.

## Methods

### Type of study

The research used a narrative exploratory design to capture the opinions and representations of the health personnel (Patton, 2002; Fortin, 2010).

### Study site

**Figure 1:**
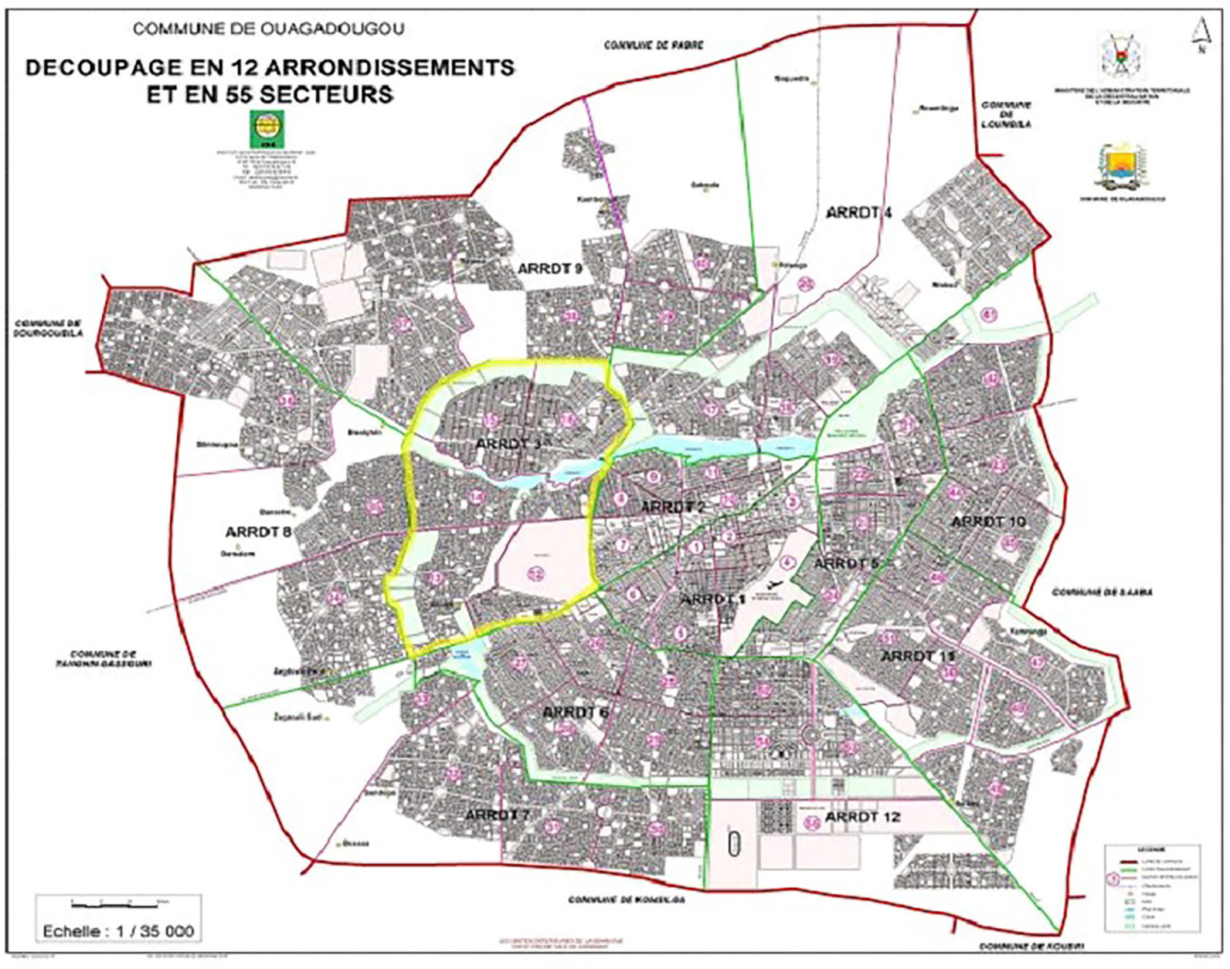
Boroughs and sectors of Ouagadougou city. Source: https://www.sig-noghin.org/wp-content/uploads/2019/03/Carte-de-Ouaga-12-arrondissements.jpg.

The research took place in Ouagadougou, the capital city of Burkina Faso. Burkina Faso is a West African country with a population of approximately twenty-one million in 2020. Ouagadougou is located in the center of the country, with a population of 2,453,496 inhabitants in 2020. It is subdivided into 12 boroughs and 55 sectors (Figure 1). The city was considered the epicenter of the epidemic in Burkina Faso because 80.81% of the total number of patients screened for COVID-19 were concentrated. As of February 10, 2022, this number was estimated at 13,841, with 171 deaths (CORUS, 2022). In addition, at the period of the study, the city concentrated the public health facilities which took in charge COVID-19 patients and few health workers received training.

Five boroughs out of twelve were selected according to their geographical location (01 Downtown, 01 East, 01 West, 01 South, 01 North). In each of the borough, a sector and then a plot were randomly selected for data collection. These are the Kamsonghin, Tanghin, Bissighin, Dassasgho, and Trame d'acceuil Ouaga 2000, respectively.

### Sampling and data collection

The target population for the research was health personnel in public and private health centers in the selected plots. In each plot, 2 health workers from a public primary health facility (Center de Santé et de Promotion Sociale) and 2 from a private health center (medical or nursing office) were randomly selected. In total, twenty (20) health workers were interviewed. The interviews were conducted in French. A bilingual social scientist member of the research team was in charge of the translation of quotes from French to English to minimize the interpretation lost. No one received specific training on COVID-19 at that period.

At the time of the data collection, none of the respondents were involved in the management of COVID-19.

Data collection took place from May 8 to 14, 2020, in the health centers selected in each plot. It was conducted by five interviewers who were trained in conducting individual interviews and in respecting barrier gestures and physical distance.

The data collection technique used was the individual in-depth interview, which was carried out using an interview guide. The interview guide was developed using existing guide for other diseases such as malaria and Ebola. The guide had nine main topics such as knowledge and perceptions of COVID-19, perceived risk of coronavirus, perceptions of care management made by the Ministry of Health, social acceptability of distancing measures, perception and social acceptability of barrier gestures, financial and social costs of preventive measures, perceptions of the actors of the response, information and communication. In the topic of knowledge and perceptions of COVID-19, there were questions related to origins and causes of COVID-19, signs, treatments, diagnosis and similarity with other diseases.

All health workers contacted agreed to participate. The interview was conducted after getting an appointment with the participant. That means it was at the convenient time, date and place for the participant. On average the interview lasted 50 min. All interviews were recorded with the consent of the respondents.

### Data analysis

All interviews were transcribed. All the transcripts were imported into QSR NVIVO 12 software. Then they were organized and coded according to a nonrigid thematic coding grid. Sections of text were labeled according to specific themes and areas of interest related to the study questions. A thematic content analysis was used to highlight categories related to the research objectives.

### Ethical issues

The overall protocol was submitted to the Health Research Ethics Committee of the Ministry of Health and received its approval (deliberation CERS N°2020-4-083). Voluntary and informed consent was requested from all participants after reading the information about the survey. All participants signed the consent form.

## Results

The study participants were 11 men and 09 women. The average age was 43 years. The providers were mainly paramedics, including 13 nurses, 02 itinerant health agents and medical personnel, including 03 doctors and 02 midwives. Among them 10 were from private sector and 10 from public one. The results are presented without any categorization because analysis per sector or type of health worker was not relevant.

The results of the analysis are about how long health workers have known about the disease, their local designation of the disease, the diseases they associate with COVID, their knowledge of the causes and origins of the disease, their knowledge of the different routes of contraction of the disease, their ability to detect symptoms of COVID 19 virus, their knowledge of the appropriate type of treatment and their experience with treatment.

### Period of awareness of the disease

One of the themes of the guide was about the first-time people had heard about COVID-19. The aim was to determine the period in which health workers learned about the disease. According to the respondents, three periods emerged: late 2019, before March 2020 and after March 2020. Majority of people (15/20) therefore were aware of the disease long before the first cases were announced in Burkina Faso which was on March 9th.

“...I can say that I have heard about COVID since the very beginning because when you follow the news, the first time I saw it was on France 24, in January, I was following the news and then we talked about it and it has been on TV since January.” (Health worker, private, 36 years old, Kamsonghin).

Few health workers only learned about it after the first cases were reported in the country as stated by one of the respondents: “Since the first case in March, when we started talking about the coronavirus in other countries at the end of February, beginning of March, but precisely here in Ouaga, it is from March 8 that I heard about it.” (Health worker, public, female, age 44, Dassasgho).

### Local name of the disease

We explored if there was any local name of the disease. In general, and according to most of respondents, there was no commonly accepted local name for the disease.

“there is no local name.” (Health worker, private, female, age 41, Tanghin).

Nevertheless, few found names in the local language because of the similarity of the symptoms with known diseases.

“Yes indeed when we talk, well, I am Mossi (Editor's note: the majority ethnic group in Burkina Faso), and when indeed we see the symptoms around us, the runny nose or sneezing frequently, and then difficulty breathing people generally say that it is because I have caught a cold and I have taken fresh air and I have traveled that it is like that some call it cold, others “*zaco*” and then the older ones speak of “*fonsré*.” (Health worker, private, male, 65, Kamsonghin).

### Similar illnesses in the community

The respondents equated COVID-19 with other diseases (based on symptoms and manifestations), particularly lung infections and respiratory diseases. Both sector provided same perceptions.

“according to the signs (...) I can say these are the signs of pneumonia.” (Health worker, private, female, 35, 06, Bissighin).

“Many diseases (huh) also have the same signs, so I can compare it to pneumonia, bronchopneumopathy.” (Health worker, public, female, age 44, Dassasgho).

Few health workers compared COVID-19 to other viral epidemics, such as Ebola, dengue, and malaria. This comparison was made because of the similarity of the signs of these diseases.

“Because it is viral, others ask “is it Ebola or dengue?”. We give them explanations saying that it is a viral disease, but COVID-19 has its specificities, i.e., headaches, respiratory problems, sore throat with cough.” (Health worker, private, male, 67 years old, Tanghin).

“...according to the signs that I see and according to the signs that we were shown, the cough, the cold, and then the fever, these signs are equivalent to the signs of malaria; often, this is what we think because the fatigue, so all that, you are constantly... you tell yourself that it is malaria that you are doing. And it's after a while that you'll see that it's not malaria, the fact that the cough persists, and then there are other digestive problems... It's almost like malaria in my opinion. Even severe malaria causes breathing difficulties and leads to death.” (Health worker, private, female, 29 years old, Dassasgho).

However, respondents qualified this by stating that the breathing difficulties associated with COVID-19 are more severe.

They said, “COVID is more dangerous than malaria. Because when it happens to the respiratory problem, it's already over for you. However, when malaria arrives at the point of distress, at least with adequate care, you can recover. But COVID, once it has reached this stage, is not as recoverable as we think.” (health worker, public, female, 41 years old, Bissighin).

### Causes and origins of the disease

Respondents are divided on the causes of COVID-19. Few said they did not know what caused it or the origin, while others attributed it to an animal or a virus.

“They say it comes from an animal.” (Health worker, private, male, 67 years old, Tanghin).

“...it's a viral disease, it's caused by a virus.” (Health worker, private, 35 years old, Bissighin).

It is important to note that all the health workers we met have general knowledge of the disease, and all agree that it is a dangerous viral disease.

“I know that the disease is due to a virus and it is a virus that is very contagious, so you can catch the disease there through the virus....” (Health worker, public, female, age 44, Dassasgho).

### Routes of transmission of COVID-19

Promiscuity, physical contact and the respiratory tract are the main routes of transmission of the disease identified by health workers of both sectors. However, they emphasize the interdependence between these different routes of transmission. Indeed, promiscuity and physical contact favor contamination because the virus is found in the body fluids of the patient (saliva, sputum) and can pass through the respiratory tract and physical contact to infect a healthy person.

“A patient with COVID can contaminate another person by sneezing, coughing and spitting....... and by breathing the air around us if there is a patient close to us, we can be contaminated.” (Health worker, private, 35 years old, female, Bissighin).

“...when someone sneezes or coughs, for example, without protecting himself, if the droplets of saliva that he emits touch the person in front of him through the upper respiratory tract, he can easily be contaminated. This is called human-to-human contamination.” (Health worker, public, male, 42 years old, Kamsonghin).

### Ability of health workers to detect symptoms of COVID-19

For all health workers, the most common symptoms of COVID-19 are runny nose, dry cough, fever, sneezing, chest pain, difficulty breathing, headache, etc. However, they recognize that these symptoms and signs alone are not enough to make a diagnosis of COVID-19.

“If I see him like this I cannot say that he has COVID 19, these are only hypotheses; he may come with a fever or a cough but it is not necessarily COVID 19, so physically you cannot see a patient and say that he has COVID 19, it is difficult. It's difficult. When you see the signs and so on, you might make a diagnosis and see if it's COVID 19 or not, but if you don't see him like that and say he has COVID 19, no, even if he starts coughing several times, it's not obvious.” (Health worker, public, female, age 44, Dassasgho).

When faced with persistent signs, they called the call center of the national center in charge of health emergency (CORUS) to have testing for COVID-19.

“It's as we go along when we see the distresses...if we see the signs of cough, cold, breathing difficulties, we call 3535....” (health worker, public, male, 54, Bissighin).

“Yes we know the symptoms, the signs such as flu, fever, sore throat as well as respiratory complications (...), but we do not confirm that it is the COVID-19 virus. It is the CORUS team that can confirm.” (Health worker, private, male, 67, Tanghin).

### Available/appropriate treatment

Several treatments were mentioned by the health workers, including modern and traditional medicine. The available treatment would be a combination of azithromycin and chloroquine mentioned by majority of health workers. Symptomatic treatment was also mentioned.

“I know that we currently use the protocol, the azithromycin and chloroquine duo with adjuvants such as vitamin C, consistent hydration and detection of fever if there is one, oxygenate the patient (…).” (Health worker, private, female, 36 years old, Kamsonghin).

“It is a symptomatic disease. Therefore, we only treated the symptoms. If a person has cough, fever or a respiratory problem, we just treat that.” (Health worker, private, female, age 41, Tanghin).

Health workers who equate COVID with “*fonsre*” or other local diseases think that traditional treatment might be appropriate.

“It is *fonsré* in Mooré (local language), but the treatment is black powder that you have to put in the porridge to drink, but that's the thing that I myself have never used.” (Health worker, private, female, 35, age 06, Bissighin).

“... perhaps the traditional people'don't call it COVID-19... but our traditional practitioners treated the same symptoms, cough, fever, sore throat, headaches. But traditionally it's not called maybe COVID 19 virus.” (Health worker, private, male, 67 years old, Tanghin).

In the search for an effective treatment against COVID-19, some health workers think of a synergy of action between modern and traditional medicine.

“At present, we are talking about the medicine from Madagascar (editor's note: covid-organics), we are talking about the medicine from Togo (editor's note: Avipirine from Benin), we are talking about the remedies of traditional practitioners. It is said that the WHO has not yet approved it, but you never know, someone may find the remedy. They say that there may be consequences, but there are people who are already using.” (Health worker, private, male, 67, Tanghin).

Nevertheless, few respondents questioned the motivations and attitudes (reservations about the efficacy of traditional African remedies) of the WHO regarding traditional medicines. They feel that in the absence of modern medicine treatment, traditional options can be supported to test for safety and offer them as treatment.

“They say that it can have consequences, but it is very easy for the WHO or for countries to check if there are toxic elements in a medicine in the laboratories. What does it cost the WHO and the states to quickly check the toxicity and validity of a drug?” (Health worker, private, male, 67 years old, Tanghin).

However in absence of reliable treatment, all health workers agreed that the best method to stop the disease is prevention, which necessarily involves respecting the measures put in place by the Ministry of health.

At the time of the data collection, all health workers said they have no experience in treating COVID-19. The experience of some was limited to treating symptoms, as illustrated by this man “we have had to treat these symptoms but not in the case of COVID-19.” (Health worker, private, male, age 41, Tanghin).

This lack of experience was due to the fact that majority of public health facilities and private health centers were not included in the response mechanism put in place by the Ministry of Health. In this sense, one of respondents stated that “we ourselves are not authorized to treat COVID-19 in our health facility.” (Health worker, public, female, age 44, Dassagho).

## Discussion

The paper presented the results of a study exploring the perceptions of health workers on COVID-19 in Burkina Faso in 2020. Specifically, we analyzed the time period during which health workers became aware of COVID-19, the local name of the disease, the diseases to which they associate COVID-19, their knowledge of the causes and origins of the disease, the different routes of transmission of the disease, the symptoms, the appropriate treatment, and their experiences with treatment. The analysis per sector (public/private) and per health worker category did not show any difference in knowledge and perceptions for COVID-19.

It should be noted, however, that none of the respondents have been involved in the management of COVID, as they do not work in management centers. These knowledge and perceptions were constructed from information received about the disease through the media.

The findings showed that the health workers interviewed had fairly good knowledge of the period of onset of the disease, which was in the first quarter of 2020. Literature on COVID-19 in Burkina Faso also places the period of knowledge of COVID-19 in the same period (Semporé et al., 2020). Similarly, the first African case of COVID-19 was reported on February 15, 2020, only two months after it was first identified in China (Sougou et al., 2020).

Health workers perceived COVID-19 as dangerous disease more than other known diseases. The article by Sougou et al. (2020) shows that Senegalese health workers have the same perceptions of the disease, especially with regard to the novelty of COVID-19, its dangerousness, and its very high frequency of infection (Abdel Wahed et al., 2020; Sougou et al., 2020).

The health workers surveyed showed good knowledge of the signs, symptoms, and possible causes of this disease. The symptoms of COVID-19 cited by the health workers were the same as those found in other studies (Abdel Wahed et al., 2020; Mahmood et al., 2020).

However, in a study conducted in Senegal, it was found that hospital staff had poor knowledge of the virus because formal information channels had not yet been set up to provide informed knowledge of the virus (Sougou et al., 2020).

One study found that risk perception is positively and significantly correlated with an index of preventive health behaviors such as hand washing, face mask wearing, and physical distancing (Dryhurst et al., 2020).

Another study conducted in Egypt further indicated that, in addition to the barrier measures mentioned, one should cough into the elbow, avoid touching the eyes, nose and mouth, avoid crowds in public places, regularly clean and disinfect surfaces, and avoid direct contact with colleagues (Abdel Wahed et al., 2020). It is true that the effectiveness of these measures also depends on their adoption and appropriation by health providers and populations (Sougou et al., 2020).

Health workers have shown that they can identify symptoms of COVID-19; therefore, as frontline workers, they can refer these suspected cases for sampling and diagnosis to the national center in charge of health emergencies (CORUS). This assumes their good knowledge of the diagnosis of the disease. Even if they seem to have a good knowledge, most of health personnel received information from TV, radio or social media (Abo et al., 2021).

Different treatments were cited by health workers. Majority cited the combination of azithromycin and chloroquine. This treatment was the one recommended by the MoH in Burkina Faso at that time. Traditional treatments were also cited by few health personnel. This was in a context of controversial public debate regarding their use (RFI, 2020). Moreover, traditional healers were asked about their involvement in the development of a treatment in collaboration with the ministry of health (Tiéné, 2020). In Africa with its long history of traditional medicine, it is crucial to investigate the efficacy of this medicine for COVID-19. This could facilitate collaboration between traditional healers and modern healers.

### Strengths and limitations of the study

There were few studies reported perceptions of health workers on COVID-19 using qualitative method in Africa. One of the strengths of this research is to provide knowledge to the existing literature on COVID-19 in Africa in the early stage of the pandemic. In fact, the research was conducted in 2020, 2 months after the first case of COVID-19 in Burkina Faso. The knowledge and perceptions of health workers reflect what they knew at that period. Currently, advanced knowledge is available, and health workers received training and update information related to COVID-19. That may have affected their knowledge and perceptions. Among the limitations of the study, there are generalization of the results. The results could not be generalized to Africa due to the context.

## Conclusion

This research explored the knowledge and perceptions of COVID-19 health workers. It generated information and knowledge that can assist in decision-making to reduce the risk of contamination of healthcare workers and promote effective protective measures. In addition the paper helps in better understanding the attitudes and management of first cases in Burkina Faso.

Research has also shown that health care workers who are well aware of the symptoms of the disease avoid diagnosing the disease outside of specialized facilities and institutions. This is especially important because the disease is still new, not very well known, and highly contagious. The results of this research open up a new and very interesting perspective, which is that of exploring the perceived risks of health care personnel in relation to COVID-19.

## Data availability statement

The datasets presented in this article are not readily available because research data are not shared. Requests to access the datasets should be directed to FYB, fadimabocoum@yahoo.fr.

## Ethics statement

The studies involving human participants were reviewed and approved by the Health Research Ethics Committee of the Ministry of Health and received its approval (deliberation CERS N°2020-4-083). The patients/participants provided their written informed consent to participate in this study.

## Author contributions

FYB and KK have contributed equally to the conception and analysis and writing of the paper. TS has contributed to the writing of the paper. AO has contributed to the analysis of the data. All authors contributed to the article and approved the submitted version.
